# Multifaceted Activities of Seven Nanobodies against Complement C4b

**DOI:** 10.4049/jimmunol.2100647

**Published:** 2022-05-01

**Authors:** Karla I. De la O Becerra, Wout Oosterheert, Ramon M. van den Bos, Katerina T. Xenaki, Joseph H. Lorent, Maartje Ruyken, Arie Schouten, Suzan H. M. Rooijakkers, Paul M. P. van Bergen en Henegouwen, Piet Gros

**Affiliations:** * Structural Biochemistry, Bijvoet Centre for Biomolecular Research, Department of Chemistry, Faculty of Science, Utrecht University, Utrecht, the Netherlands;; † Cell Biology, Neurobiology & Biophysics, Department of Biology, Faculty of Science, Utrecht University, Utrecht, the Netherlands;; ‡ Membrane Biochemistry and Biophysics, Bijvoet Centre for Biomolecular Research, Department of Chemistry, Faculty of Science, Utrecht University, Utrecht, the Netherlands; and; § Medical Microbiology, University Medical Center Utrecht, Utrecht, the Netherlands

## Abstract

Cleavage of the mammalian plasma protein C4 into C4b initiates opsonization, lysis, and clearance of microbes and damaged host cells by the classical and lectin pathways of the complement system. Dysregulated activation of C4 and other initial components of the classical pathway may cause or aggravate pathologies, such as systemic lupus erythematosus, Alzheimer disease, and schizophrenia. Modulating the activity of C4b by small-molecule or protein-based inhibitors may represent a promising therapeutic approach for preventing excessive inflammation and damage to host cells and tissue. Here, we present seven nanobodies, derived from llama (*Lama glama*) immunization, that bind to human C4b (*Homo sapiens*) with high affinities ranging from 3.2 nM to 14 pM. The activity of the nanobodies varies from no to complete inhibition of the classical pathway. The inhibiting nanobodies affect different steps in complement activation, in line with blocking sites for proconvertase formation, C3 substrate binding to the convertase, and regulator-mediated inactivation of C4b. For four nanobodies, we determined single-particle cryo-electron microscopy structures in complex with C4b at 3.4–4 Å resolution. The structures rationalize the observed functional effects of the nanobodies and define their mode of action during complement activation. Thus, we characterized seven anti-C4b nanobodies with diverse effects on the classical pathway of complement activation that may be explored for imaging, diagnostic, or therapeutic applications.

## Key Points

- Diverse binding properties are revealed for seven nanobodies against C4b.
- Cryo-electron microscopy structures of C4b–nanobody complexes indicate nanobodies’ modes of action.
- Nanobodies have therapeutic potential and are useful for labeling studies.

## Visual Abstract

**Figure:**
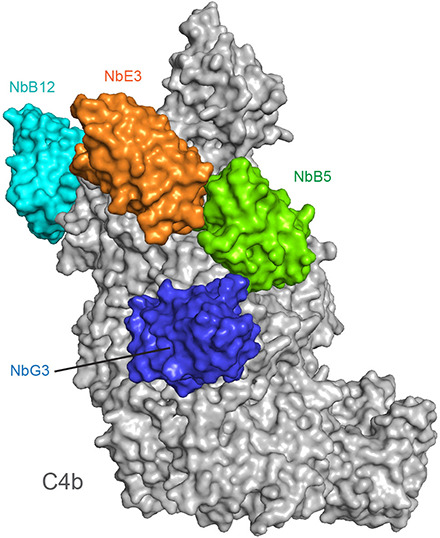

## Introduction

Mammalian complement clears invading microbes, immune complexes, and altered host cells from blood and interstitial fluids (1). The complement system consists of multiple proteins that form a tightly controlled proteolytic cascade. Uncontrolled activation of this cascade contributes to various inflammatory, autoimmune, and neurodegenerative disorders, such as systemic lupus erythematosus, glaucoma, Alzheimer disease, and schizophrenia (2–8). Hence, diverse approaches to modulate complement activation for therapeutic applications are currently in development (9).

The complement cascade consists of three main pathways: the classical, lectin, and alternative pathways (CP, LP, and AP, respectively) (10, 11). Both the CP and LP are initiated upon multivalent recognition of danger patterns on surfaces by large protease complexes, C1 for the CP and lectin mannose-associated serine-protease complexes for the LP. Activation of these complexes triggers the proteolytic complement cascade by cleaving complement protein C4 (12, 13). C4 is cleaved into a small fragment, C4a, and a large fragment, C4b, that act as an opsonin. C4b is covalently linked to the targeted surface through a reaction of its reactive thioester moiety with available nucleophiles on the surface (14). Subsequently, C4b binds protease C2 and forms proconvertase C4b2 that is further processed into C4b2b, which is the (CP and LP) C3 convertase that cleaves C3 (15). C3, which is homologous to C4, is cleaved into anaphylatoxin C3a and opsonin C3b, which is deposited onto a surface similar to C4b. C3b deposition initiates the AP amplification loop by assembling the AP C3 convertase (C3bBb) (16). Eventually, both C3 convertases trigger the terminal pathway through switching convertase specificity from C3 to C5 and initiating formation of anaphylatoxin C5a and membrane attack complexes (17). In short, activation of the complement pathways results in inflammatory responses through anaphylatoxins, phagocytosis through C4b and C3b opsonization, and cell lysis though pore formation in cell membranes (2, 18).

C4 is a 204 kDa protein (formed by three polypeptide chains, β-α-γ, with m.w. of 75, 93, and 33 kDa, respectively) that circulates in serum at concentrations of ∼3 µM (19). C4 contains eight macroglobulin (MG) domains, an anaphylatoxin-like C4a domain, a C1r/C1s, Uegf, Bmp1 (CUB) domain, a thioester-containing domain (TED), and the C-terminal C345c (CTC) domain (20), similar to its structural and functional homolog C3 (21). Upon activation, the C4a domain (9 kDa) is removed, and the remaining C4b fragment (195 kDa) undergoes large conformational changes. Domains MG7-8, CUB, and TED rearrange, and changes within TED expose and activate the previously buried thioester moiety for covalent attachment to surfaces (19, 22). The N-terminal region formed upon removal of the anaphylatoxin-like C4a domain, denoted α′-chain N-terminal region (α′NT), relocates from MG2 and MG3 to MG7 on the opposite side of the molecule. Based on the overall homologies of C4b and C3b and of proteases C2 and factor B, MG2, MG6, MG7 with bound α′NT, CUB, and CTC of C4b are expected to form a composite interaction site for C2 binding in the formation of the CP and LP C3 proconvertase, similar to what is observed for the AP C3 proconvertase (C3bB) (19, 23, 24). Next, the N-terminal domains of C2 are removed by proteolysis, yielding C4b and the proteolytic fragment C2b, the CP and LP C3 convertase (C4b2b); the nomenclature of C2 fragments, C2a and C2b, is according to the International Complement Society and European Complement Networks (25). A putative model for C3 substrate binding to C3 convertases is based on a structure of the AP convertase C3bBb stabilized by *Staphylococcus aureus* SCIN, which revealed a structural dimer that suggested a C3b-C3 arrangement for the C3bBb convertase to bind substrate C3 (26). This model explains the mechanism of inhibition by compstatin (27) and complement receptor of the Ig superfamily, CRIg (28), and is supported by disease-related mutations (29, 30). Soluble regulators, C4b-binding protein (C4BP) and factor H (FH) with decay-accelerating activity and cofactor activity for factor I (FI) mediate cleavage and promote convertase dissociation and degradation of C3b into iC3b and C4b into C4c and C4d. These degradation products are inactive with respect to convertase subunit activity (31–33). Regulators presented on host cell surfaces, such as decay-accelerating factor (DAF), membrane cofactor protein (MCP), and complement receptor 1 (CR1), provide direct protection to host cells (34). For the homologous protein C3b, detailed structural data are available that define the binding of regulators, FH, DAF, MCP, and CR1, as well as protease FI to C3b (35–37). The functional and structural data provide a framework for correlating activities and binding sites of anti-C4b nanobodies.

Currently, no specific drugs to regulate initiation through the CP or LP in complement-related diseases have been approved (9), although, similar to our work, nanobodies with inhibitory properties have been developed against C1q, C3, and C4b (38–41). With respect to C5 inhibition, in addition to mAbs (42, 43), it has recently been shown that inhibition can be achieved with small domain peptides derived from bovine Abs that are stable enough on their own to act as Ag-binding entities, denominated “knobs” (44). The development of small high-affinity binding molecules, such as nanobodies and knobs, opens the possibility to generate low-immunogenicity and highly stable protein fragments suitable for functional studies and for the design of therapeutic strategies (45). Here, we present a set of seven high-affinity nanobodies raised against C4b. Functional and structural analyses provided insights into the modes of action of the nanobodies and identified inhibitors that block different steps of the complement pathway. Furthermore, we demonstrate the applicability of one noninhibitory nanobody for use in fluorescence microscopy of erythrocyte lysis by labeling deposited C4b and imaging the effective modulation of the CP by one of the nanobody inhibitors.

## Materials and Methods

### Llama immunization and nanobody library construction

Llamas (*Lama glama*) were immunized with purified human C4b (Complement Technologies) to generate a C4b nanobody immune library. Five doses of 25 μg of C4b per dose were administered s.c., over a 10-wk period. C4b-specific immune responses were confirmed by ELISA using preimmunization sera as well as sera after the first two boosters. After completion of the immunization scheme, total RNA was extracted from PBLs of the immunized llamas (Eurogentec Nederland B.V.). An immune variable domain of the H chain from H chain–only Ab (VHH) library was constructed as described before (46). In brief, total RNA was transcribed to cDNA by RT-PCR. The purified cDNA was used as a template for VH gene amplification and the introduction of a SfiI restriction site at the 5′ end of VHH encoding sequences using a two-step nested PCR. The purified repertoire of VHH genes was digested with SfiI-BstEII, ligated into pUR8100 phagemid vector, and transformed into electrocompetent *Escherichia coli* TG1 bacteria yielding a nanobody M13 phage display library size of 7 × 10^6^.

### Nanobody selection

For C4b nanobody selection, a nanobody library was panned on purified C4b in PBS and immobilized on streptavidin-coated plates. To this end, C4b at a concentration of 5.1 µM was incubated with a 40× molar excess of EZ link Maleimide-PEG2-Biotin (Thermo Fisher Scientific, 210901BID) in PBS, pH 7.4, overnight at 4°C. Unreacted linker was separated with a Bio-Spin 6 column (Bio-Rad Laboratories) that was equilibrated in PBS. Plates were coated with 1 µg of biotinylated C4b per streptavidin‐coated well (Pierce Streptavidin‐Coated High‐Capacity Plates, 15501; Thermo Fisher Scientific) after incubation at 4°C for 16 h in PBS. The immobilized target was panned with 7 × 10^9^ phages preblocked with 2% (w/v) milk for 2 h at room temperature (RT), followed by several 0.05% (v/v) Tween 20/PBS and PBS washes. Remaining bound phages were eluted by incubation of the wells with 0.1 M triethylamine, pH 12.0, for 30 min at RT while shaking. Eluates were then neutralized by addition of 1 M Tris-HCl, pH 7.4, before reinfection of TG1 bacteria. The resulting enriched phage library was submitted to a second round of selection. From the second selection round output, 94 single colonies of TG1 bacteria were picked and screened on streptavidin-immobilized C4b plates following a previously described phage ELISA protocol (47).

### Nanobody production and purification

Phagemids of positive clones were isolated and sequenced, and unique nanobody sequences were subcloned into a modified pHEN6-thrombin cleavage site 6xHis vector (gift from Dr. F. Opazo, University of Göttingen Medical Center, Göttingen, Germany), containing an N-terminal pelB leader sequence for periplasmic secretion of produced nanobodies. Nanobodies were produced in *E. coli* BL21 Codon Plus (DE3)-RIL bacteria strain (Agilent Technologies). Bacteria were grown in 2× yeast tryptone media supplemented with ampicillin to a final concentration of 100 μg/ml (Sigma‐Aldrich BV) at 37°C while shaking. When the cultures reached an OD_600_ of 0.6, nanobody expression was induced with 1 mM isopropyl β-d-1-thiogalactopyranoside (Thermo Fisher Scientific). Bacteria were grown overnight at 25°C, and cells were collected and resuspended in ice-cold PBS. Suspended cells were submitted to two freeze–thawing cycles, and the supernatant (periplasmic fraction) was collected after centrifugation at 5000 × *g* for 20 min at 4°C. Periplasm was incubated with 0.5 ml/L culture of Ni‐NTA beads (Qiagen) for 2 h while rotating at 4°C. The beads were placed in empty gravity flow columns (Bio-Rad Laboratories) and washed with 30 column volumes (CV) of buffer A (20 mM HEPES, pH 8.0, 500 mM NaCl) followed by a wash with 10 CV of buffer A supplemented with 20 mM imidazole and eluted with 2 CV of buffer B (20 mM HEPES, pH 8.0, 500 mM NaCl, 250 mM imidazole). The nanobodies were concentrated in Amicon Ultra 4-ml centrifugal filters with a membrane cutoff of 3000 Da. Finally, the nanobodies were subjected to a size exclusion chromatography (SEC) purification step through injection into a Superdex 75 column (GE Healthcare) preequilibrated with SEC buffer (20 mM HEPES, pH 7.4, 150 mM NaCl).

### Surface plasmon resonance (SPR)

C4b was biotinylated as mentioned in the *Nanobody selection* section. The nanobodies NbE11, NbH11, NbB5, and NbB12 were biotinylated by incubating them with EZ-link NHS-Peg4-Biotin (Thermo Fisher Scientific) for 16 h at 4°C. Subsequently, the free linker was removed with a Bio-Spin 6 column preequilibrated with PBS. The biotinylated C4b (50 nM) and nanobodies (100 nM) were spotted on a planar streptavidin-coated chip (P-strep, Sens BV) under a continuous flow for 1 h using a continuous flow microspotter (Wasatch). All SPR experiments were performed on the IBIS-MX96 (IBIS Technologies) in SPR buffer (20 mM HEPES, pH 7.4, 150 mM NaCl, and 3 mM MgCl_2_). For the determination of C4b affinities of NbE3, NbE11, NbG3, and NbH9, the nanobodies were injected in a 14-step twofold dilution series on a C4b-coated surface. After each injection, the surface was regenerated using SPR buffer supplemented with 2 M NaCl for NbG3 and NbE3 or SPR buffer supplemented with 1 M MgCl_2_ for NbH9 and NbE11. In the experiment with NbB5, NbB12, NbE11, and NbH11 coated on the surface, kinetic titration was performed by injecting C4b in a five-step twofold dilution range without any regeneration. The last dissociation step was 1 h for a reliable determination of the *k*_off_. The binding kinetics were determined using Scrubber 2.0 (BioLogic Software). The data were fitted with a simple bimolecular model except for the nanobodies NbE3, NbE11, and NbH11, when they were used as a ligand, of which the kinetics were determined by fitting them with a model with mass transport–limited interactions. For one-to-one nanobody competition assays, single nanobodies were injected sequentially (100 nM) followed by their simultaneous injection on the C4b-coated surface.

### SEC assay for proconvertase formation and C4 nanobody binding

C4b alone or C4b mixed with C2 (Complement Technologies) in a 1:1 molar ratio was incubated for 5 min on ice followed by the addition of each nanobody in a 1:1.2 molar ratio of C4b to nanobody, respectively. To evaluate nanobody binding to C4 by SEC, nanobodies were previously coupled to Alexa Fluor 488 NHS Ester (Thermo Fisher Scientific) by incubating the nanobody with a fourfold molar excess of fluorophore for 1 h at RT in the dark. Excess fluorophore was removed with a Bio-Spin 6 column preequilibrated in SEC buffer. Fluorescent nanobody was mixed with C4b or C4 (Complement Technologies) in a 2:1 molar ratio, respectively. Samples were injected in a Superdex 200 Increase column (GE Healthcare) preequilibrated in SEC buffer connected to an HPLC system (Shimadzu). Samples were monitored by absorbance (280 nm) and fluorescence SEC (excitation 494 nm, emission 517 nm).

### Classical pathway hemolytic assay

Sheep blood (Alsever Biotrading) was washed twice with PBS to obtain pelleted RBCs (SRBCs). Normal human serum was first preabsorbed with pelleted SRBCs (for 15 min on ice) to remove naturally occurring Abs that react with the “Forssman” glycosphingolipids present on SRBCs (48). The cell pellet was discarded, and the preabsorbed serum was used as a complement source in the total complement hemolytic activity. For the total complement hemolytic activity experiment, a new SRBC pellet was washed and resuspended in Veronal-buffered saline (VBS) containing 0.5 mM CaCl_2_ and 0.25 mM MgCl_2_ (VBS^++^). SRBCs at a concentration of 2 × 10^8^ cells/ml were coated with specific Ab by incubation with anti-SRBC IgG (Diamedex) to a final dilution of 2.5 μg/ml for 10 min at RT. Cells were further washed and resuspended in a smaller volume of VBS^++^ to achieve a final concentration of 2 × 10^8^ cells/ml for the assay. In a round-bottomed 96-well plate, 20 µl IgG-opsonized SRBCs and 20 µl of preabsorbed human serum (ranging from 2.5% to 10% [v/v]) were mixed with 20 µl of buffer and 1 μM nanobody or, for controls, 10 μg/ml of mAb anti-C1q ATCC HB8327 4a4b11, mAbC1q (produced in house Utrecht Medical Center), or 10 mM EGTA final concentrations. Mixtures were incubated for 30 min at 37°C on a shaking plateau. Maximum lysis control was established by lysing the cells with MilliQ water. After centrifugation, release of hemoglobin was measured at 405 nm after transferring 30 µl of supernatant to a half-area flat-bottomed plate containing 60 µl of MilliQ per well. The percentage of lysis was calculated with the following formula: % lysis = ([Sample OD_405_ – Sample Zero OD_405,_ no serum]/[cell water lysis OD_405_ – Zero OD_405_, cells in VBS^++^]) × 100, with a total of five replicates per sample. Statistical analysis was performed as one-way ANOVA followed by Dunnett’s multiple comparisons test using GraphPad Prism version 9.

### C4b proteolytic inactivation assay by C4BP and FI

C4b (1 μM) was mixed with each nanobody variant in a 1:2 molar ratio, respectively, and incubated for 5 min at RT in SEC buffer. C4BP and FI (Complement Technologies) were added in a 0.1:1 and 0.05:1 molar ratio or 0.5:1 and 0.05:1 compared with C4b, respectively. The reaction mixtures and pure proteins as controls were incubated for 1 h at 37°C and then run on SDS-PAGE gels for analysis.

### Cryo-electron microscopy

C4b (1 μM) was incubated with nanobody (NbB5, NbB12, NbE3, or NbG3) in PBS at a 1:2.2 molar ratio on ice for 1 h before grid freezing, resulting in final protein concentrations ranging from 0.24 to 0.26 mg/ml measured by NanoDrop. C4b–nanobody complex (2.8 µl) was pipetted onto glow-discharged R1.2/1.3 200-mesh Au holey carbon grids (Quantifoil) and then plunge frozen in liquid ethane with a Vitrobot Mark IV (Thermo Fisher Scientific) at 4°C.

All cryo-electron microscopy (cryo-EM) data were collected on a 200-kV Talos Arctica microscope (Thermo Fisher Scientific) equipped with a K2 summit detector (Gatan) and a post column 20-eV energy filter. Videos were collected in counting mode using EPU (Thermo Fisher Scientific) at 130,000× magnification with a pixel size of 1.0285 Å/pixel. For each dataset, videos were collected in 30–34 frames with a total electron exposure of 50–55 e-/Å2 (measured in an empty hole without ice). The defocus values ranged from −0.8 to −3.0 µm.

The collected micrographs were processed in the pipeline of RELION version 3.1 (49, 50). Beam-induced motion and the camera gain were corrected using MotionCor2 (51), and contrast transfer function (CTF) spectra were estimated in GCTF (52) or CTFFIND4 (53). For each separate dataset, particle picking was first performed using the NeuralNet particle picker in EMAN2 (54). The particle coordinates were imported in RELION, and the particles were extracted and subjected to two-dimensional (2D) classification. The 2D class averages containing secondary structure details were then used for automated particle picking in RELION (55). This resulted in 522,079 particles for the C4b-NbB5 dataset, 602,481 particles for the C4b-NbB12 dataset, 617,600 particles for the C4b- NbE3 dataset, and 845,087 particles for the C4b-NbG3 dataset. The particles were then binned three times (resulting pixel size 3.09 Å).

For the C4b-NbB5, C4b-NbB12, and C4b-NbE3 datasets, the particles were subjected to one round of 2D classification, removing 930 junk particles for C4b-NbB5, 33,589 junk particles for C4b-NbB12, and 83,853 junk particles for C4b-NbE3. The remaining particles were subjected to a 3D classification into five classes, using as the initial model a 40 Å filtered map constructed through one of the C4b molecules in PDB accession no. 4XAM (19). For each dataset, a single good class that displayed features of nanobody-bound C4b could be distinguished. The particles belonging to the good class (176,318 for C4b-NbE3, 275,925 for C4b-NbB5, and 185,116 for C4b-NbB12) were then unbinned (pixel size 1.03 Å, box size of 300 pixels) and 3D autorefined, yielding a map at 4.90 Å for C4b-NbB5, 6.17 Å for C4b-NbB12, and 5.05 Å for C4b-NbE3. A post-processing step, in which the density maps were masked, improved the resolution of the maps to 4.2 Å for C4b-NbB5 and C4b-NbE3 and to 4.4 Å for C4b-NbB12. Next, CTF refinements were performed to correct for higher-order aberrations and anisotropic magnification present in each dataset and to estimate per-particle defocus values (50). The particles were then subjected to Bayesian polishing, followed by a 3D autorefinement. This workflow of CTF refinements, Bayesian polishing, and 3D autorefinement was repeated –two or three more times. The final 3D autorefinements with a soft mask and solvent-flattening Fourier shell correlations (FSCs) yielded a density map at a global resolution of 3.43 Å for C4b-NbB5, 3.76 Å for C4b-NbB12, and 3.39 Å for C4b-NbE3, according to the gold standard FSC = 0.143 criterion (56).

To further improve the quality of the NbB12 density in the C4b-NbB12 map, a soft mask was created around NbB12 and the MG8 domain of C4b. This mask was used for particle subtraction, and all density outside of the mask was subtracted. Then, the subtracted particles were 3D classified without image alignment (tau2_fudge 20) into five classes. The 128,161 particles belonging to the class with the most secondary structure details were reverted back to the original full-size particles. These particles were subjected to a 3D autorefinement with a soft mask and solvent-flattening FSCs and yielded a density map of C4b-NbB12 at a global resolution of 3.76 Å. The removal of particles following the procedure of particle subtraction and 3D classification without image alignment did not affect the global resolution of the map but slightly improved the density quality at the C4b–NbB12 interface.

The C4b-NbG3 dataset suffered from severe particle clustering and from preferred particle orientation. Preliminary processing in RELION with particles picked through EMAN2 yielded a refined density map at 8.3 Å resolution before masking. As stated above, 2D class averages obtained through this preliminary processing step were used as templates for autopicking in RELION. These 845,087 RELION-picked particles were subjected to two rounds of 2D classification, and 68,544 junk particles were removed. To overcome issues related to particle clustering, the remaining particles were 3D classified into three classes with a soft mask. The soft mask was created using the previous 8.3 Å C4b-NbG3 map as a template. The 207,769 particles belonging to the least distorted class that displayed features of nanobody-bound C4b were selected and unbinned (pixel size 1.03 Å, box size of 300 pixels). A 3D autorefinement of these particles yielded a density map at 6.7 Å, and a post-processing step in which the map was masked improved the resolution to 4.54 Å. Next, two cycles of CTF refinement, Bayesian polishing, and a 3D autorefinement were performed. The final 3D autorefinement with a soft mask and solvent-flattening FSCs yielded a density map of C4b-NbG3 at a global resolution of 3.96 Å according to the gold standard FSC = 0.143 criterion.

### Model building and refinement

To build the models of C4b-NbB5, C4b-NbB12, and C4b-NbE3, the C4b molecule comprising chains D, E, and F of PDB accession no. 5JTW (57) was rigid body fitted into the cryo-EM maps. Starting models for the three nanobodies were generated through the SWISS-MODEL server (58) based on the nanobody sequences. These models were also rigid body fitted in the cryo-EM maps, and the CDRs of the nanobodies were manually rebuilt in Coot (59). The three models were then iteratively refined using Coot (manually) and using Phenix real-space refine (60) with geometric restraints. C4b was modeled based on the sequence of C4-A. The CTC domain of C4b was not included in the final structures because of the weak density for this domain in the C4b-NbB5, C4b-NbB12, and C4b-NbE3 maps. Model building in Coot was facilitated by cryo-EM maps that were post-processed using DeepEMhancer (61). These DeepEMhancer C4b-nanobody post-processed maps were obtained through the COSMIC2 web platform for cryo-EM data analysis (62).

Figures that depict protein structures were prepared using Pymol (Schrödinger). Cryo-EM density figures were created using UCSF Chimera (63) and UCSF ChimeraX (64).

### C4b deposition and lysis of SRBCs

SRBCs were washed twice and further diluted to 5 × 10^6^ cells/ml in Ringer buffer (125 mM NaCl, 5 mM KCl, 1 mM MgSO_4_, 5 mM glucose, 1 mM CaCl_2_, and 32 mM HEPES/NaOH, pH 7.4). μ-Slide 8-well chamber slides (IBIDI) were preincubated with 1 mg/ml BSA for 1 h at 4°C and washed once with Ringer buffer. The cell suspension was transferred into the wells. SRBCs were observed on a Zeiss LSM880 confocal microscope with a 100× oil immersion objective. NbB12 was coupled to Alexa Fluor 488 NHS ester as previously stated for SEC assays (NbB12-AF488). Cell sulforhodamine B influx and NbB12-AF488 binding were observed at 560/566–685-nm and 488/493–566-nm excitation/emission, respectively. Morphology of SRBCs was followed through the transmission channel upon excitation at 488 nm. No bleed-through from the sulforhodamine toward the Alexa Fluor 488 channel was observed at working conditions and excitation power. Different reagents were added to the cells sequentially in the following order and concentrations. First, sulforhodamine B and NbB12-AF488 were added at 40 and 0.6 µM, respectively, and the suspension was gently homogenized. SRBCs were first checked for sulforhodamine B influx and NbB12-AF488 binding before 5% human serum (normal or C4 depleted; Complement Technologies) or inhibitory nanobody NbE3 at a final concentration of 1 µM was added. At this stage, cells were incubated for at least 15 min and monitored for permeabilization or NbB12–Alexa Fluor 488 surface binding. To start the complement cascade, anti-SRBC IgG was added to a final dilution of 1:4000, and NbB12–Alexa Fluor 488 deposition and permeabilization were followed for 30 min. In C4-depleted media, C4 was added 15 min after the addition of anti-SRBC IgG and followed for 30 min.

### Data availability

The model coordinates and cryo-EM density maps of the structures have been deposited under the following accession numbers to, respectively, the Protein Data Bank (PDB) and the Electron Microscopy Data Bank (EMDB): PDB 7B2P and EMD-11989 for the C4b-NbB5 complex, PDB 7B2Q and EMD-11990 for the C4b-NbB12 complex, PDB 7B2M and EMD-11988 for the C4b-NbE3 complex, and EMD-11991 for the C4b-NbG3 complex. The EMDB depositions include unfiltered half maps, nonsharpened unmasked maps, and sharpened masked maps. The model coordinates and cryo-EM density maps can be found at the following website addresses for the PDB (http://www.rcsb.org/) and the EMDB (http://www.ebi.ac.uk/emdb/).

## Results

### Nanobody generation and binding analyses

**FIGURE 1.**
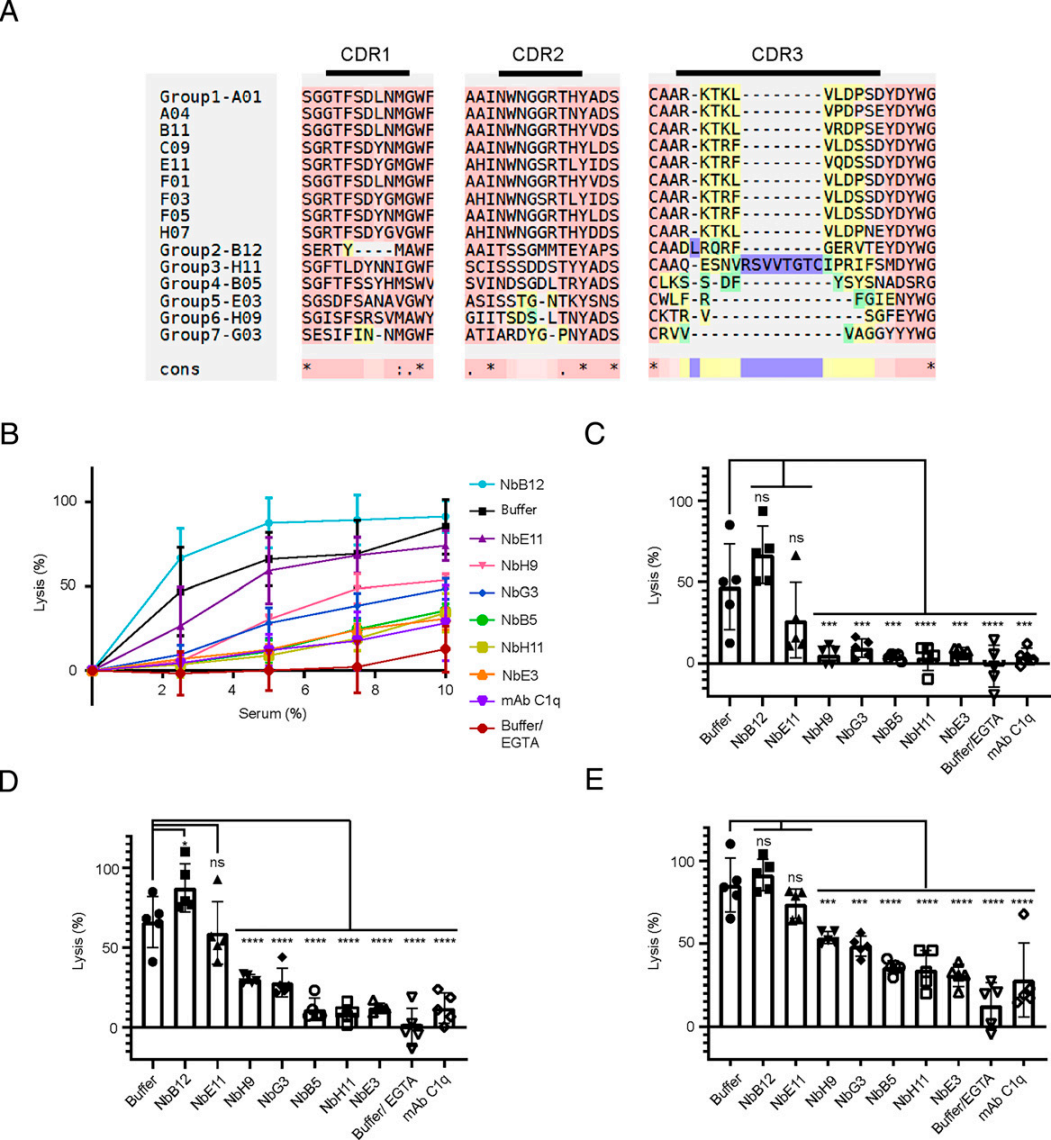
Nanobody CDR sequence comparison and impact of anti-C4b nanobodies on complement lysis of SRBCs. (**A**) Sequences were allocated in groups of families (groups) according to sequence homology (73). Groups 2–7 contained a single sequence, whereas group 1 contained 9. NbE11 was selected from group 1 for further analysis. Conserved sequences are shown in dark red, and total amino acid homology between sequences is marked with an asterisk symbol in consensus (cons). Light red denotes small variation in amino acids between overall alignment. CDR3 (most V region) is color coded from dark red (homology) to yellow, green, and finally purple denoting higher variability. (**B**) Classical pathway-dependent hemolysis of SRBCs in the presence or absence of nanobodies (1 µM) at human serum concentrations ranging from 2.5% to 10% (v/v). Buffer control indicates lysis of erythrocytes in the absence of inhibitors. A mAb against C1q (mAbC1q) and 10 mM EGTA were included as controls for CP-specific hemolysis inhibition. Lysis of SRBCs at (**C**) 2.5%, (**D**) 5%, and (**E**) 10% serum were also represented in bars for clarity and analyzed by one-way ANOVA against sample with no nanobody (buffer) at the respective serum concentrations (*n* = 5), where ns denotes *p* > 0.05 and *, ***, and **** denote statistical significance of *p* ≤ 0.05, *p* ≤ 0.001, and *p* ≤ 0.0001, respectively.

After immunizing a llama with purified human C4b, 15 positive nanobody clones were identified, sequenced, and clustered into 7 different families (Fig. 1A). One nanobody per family was chosen for further experiments: NbB5, NbB12, NbE3, NbE11, NbG3, NbH9, and NbH11. Nanobody samples of high purity were characterized for their interaction with C4b and their ability to modulate complement activation.

Binding affinities for C4b of the seven nanobodies were determined using SPR (Table I, Supplemental Fig. 1A–H). At first, we immobilized C4b on the SPR-streptavidin chip through a biotinylated linker coupled to its thiol group, mimicking the orientation of C4b deposited on surfaces. This yielded affinities of 3.2–0.17 nM for NbE3, NbE11, NbG3, and NbH9 (Table I). Because no dissociation from the chip was observed for NbB5, NbB12, and NbH11 and no suitable regeneration buffer was found, we used kinetic titration experiments to determine their affinity for C4b. NbB5, NbB12, and NbH11 (and additionally NbE11) were immobilized on the SPR chip, and C4b was used as an analyte. This resulted in *K*_D_ values of 14–67 pM for NbB5, NbB12, and NbH11 (Table I), whereas comparable *K*_D_ values were found for NbE11 of 0.17 and 0.21 nM using both setups. Furthermore, we used SPR with C4b-coated chips and sequential nanobody injection to test whether nanobodies overlapped in their C4b binding sites. Most nanobodies could interact with C4b simultaneously, except for the pairs NbB5-NbH11 and NbH9-NbE11, which showed nonadditive responses in SPR, indicative of overlapping binding sites (Supplemental Fig. 1I–Q). In summary, the seven nanobodies bind C4b with very high affinities ranging from 3.2 nM to 14 pM at five unique binding sites.

**Table I.** Binding affinities of seven nanobodies for C4b

Nanobody	*K*_m_ (m^−1^ s^−1^) × 10^8^	*K*_a_ (m^−1^ s^−1^) × 10^6^	*K*_d_ (s^−1^) × 10^−3^	*K*_D_ (nM)
NbE3	1.3 ± 0.300	21 ± 20.000	6.6 ± 0.700	0.318 ± 0.010
NbE11	—	2.0 ± 0.050	0.33 ± 0.050	0.166 ± 0.020
NbG3	—	0.82 ± 0.003	2.6 ± 0.010	3.2 ± 0.100
NbH9	—	3.4 ± 0.600	0.58 ± 0.030	0.177 ± 0.040
NbB5	—	0.20 ± 0.020	0.013 ± 0.0004	0.067 ± 0.006
NbB12	—	0.28 ± 0.020	0.0038 ± 0.0004	0.014 ± 0.004
NbE11^*a*^	4.10 ± 0.600	1.0 ± 0.010	0.22 ± 0.010	0.21 ± 0.007
NbH11	0.94 ± 0.100	3.0 ± 0.200	0.17 ± 0.004	0.57 0.002

Nanobody affinities of NbG3, NbE3, NbE11, and NbH9 were determined by SPR with C4b as ligand on the SPR chip. Affinities of NbB5, NbB12, NbE11, and NbH11 were determined using C4b as analyte and nanobodies as a ligand. The affinity of NbE11 for C4b was established through both approaches. All measurements were performed in duplicate.

a Denotes that NbE11 was used as a ligand.

Next, we tested whether the seven nanobodies also bind C4. Complexes of nanobodies with either C4 or C4b showed minimum peak shift in SEC from C4 or C4b elution profiles; therefore, fluorescence of labeled nanobodies was monitored at the elution volume of C4 and C4b (Supplemental Fig. 2A–G). Only NbE11 and NbG3 showed comparable peak heights in the presence of C4 and C4b, indicating that these nanobodies exhibit similar affinities to C4 and C4b (Supplemental Fig. 2A, B). Incubation of C4 with NbB5, NbB12, NbE3, NbH9, or NbH11 resulted in a reduced height of the C4 elution peak, corresponding to diminished affinities of these nanobodies for C4 compared with C4b (Supplemental Fig. 2C–G). These data indicate that five of seven nanobodies (NbB5, NbB12, NbE3, NbH9, and NbH11) have a preferential binding mode for C4b compared with C4, whereas two nanobodies (NbE11 and NbG3) bind both C4 and C4b with similar affinity.

### Modulation of complement activation

We next assessed whether the seven nanobodies modulated complement activity in a classical pathway hemolytic lysis assay. Ab-sensitized SRBCs were incubated with normal human serum (2.5–10% [v/v]), and complement-mediated lysis of erythrocytes in the presence or absence of nanobodies was quantified by released hemoglobin (65) (Fig. 1B–E). NbB12 did not reduce lysis, and NbE11 reduced it minimally. Reduced lysis indicating complement inhibition was observed for (in decreasing order) NbE3, NbH11, NbB5, NbG3, and NbH9 at all serum concentration tested (*p* > 0.05), whereas the reduced lysis observed for NbE11 was not significant (*p* < 0.05), though four of five data points indicated an inhibitory effect at 2.5% and 5% serum (Fig. 1C, D). NbH9 and NbG3 strongly reduced lysis at 2.5% and 5% serum (Fig. 1B–D), but were less potent at a serum concentration of 10% (Fig. 1B, E). NbB5, NbE3, and NbH11 significantly reduced lysis at every tested serum concentration, and inhibition was comparable to that of the controls, 10 mM EGTA or a mAb blocking C1q (mAbC1q). We conclude that three of seven nanobodies (NbB5, NbE3, and NbH11) are strong inhibitors and two (NbH9 and NbG3) are moderate inhibitors, whereas two other nanobodies either did not inhibit (NbB12) or minimally inhibited (NbE11) complement activation.

### Blocking proconvertase formation

**FIGURE 2.**
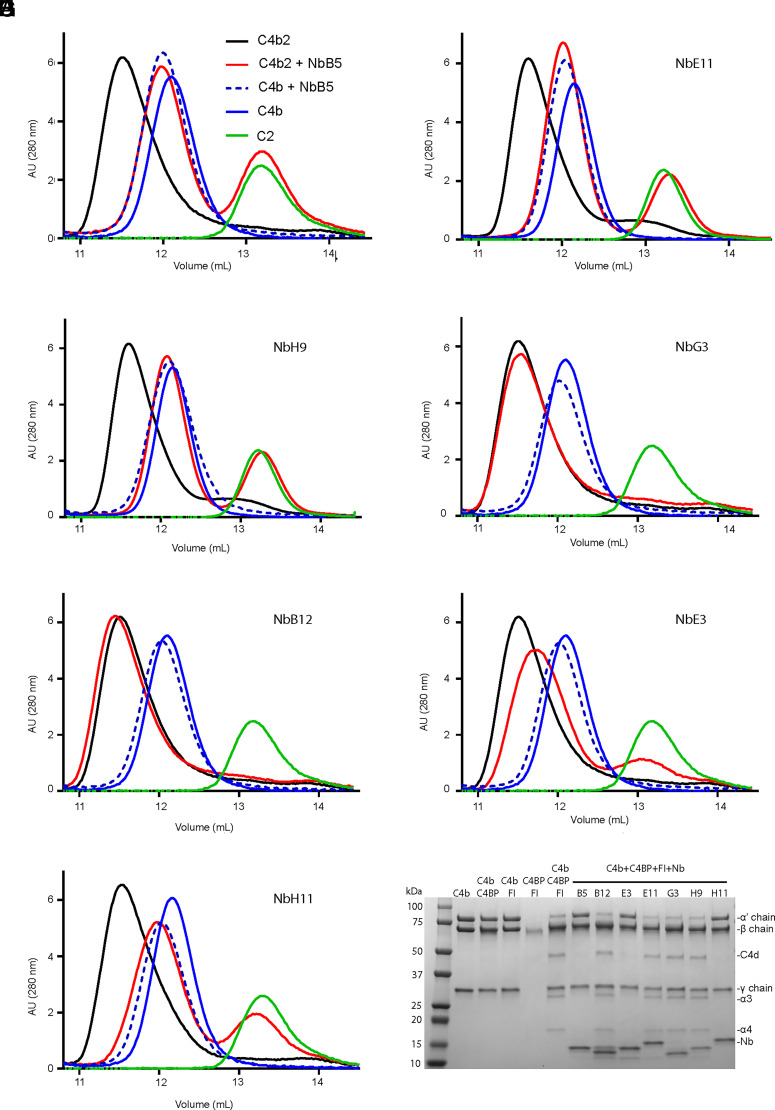
Nanobody effects on the association of C4b with protein partners. SEC elution profiles of C4b (blue line), C2 (green line), the C4b2 complex (black line), C4b–nanobody complex (blue dotted line), and C4b2 (red line) in the presence of (**A**) NbB5, (**B**) NbE11, (**C**) NbH9, (**D**) NbG3, (**E**) NbB12, (**F**) NbE3, and (**G**) NbH11. An increase in elution volume of C4b2 in the presence of nanobodies (red line) indicates that C4b2 complex formation is inhibited. (**H**) C4BP- and FI-mediated C4b cleavage of C4b into C4c and C4d in presence of nanobodies, assessed by SDS-PAGE. Cleavage is monitored through the disappearance of the C4b α′chain (87 kDa) and the formation of its breakdown products C4d (47 kDa), α3 (25 kDa), and α4 (17 kDa) at 37°C for 1 h at a molar ratio 1:0.1:0.02:2 of C4b:C4BP:FI:nanobody, respectively. Samples of (A)–(H) were run in duplicate, but only one run result is shown per sample for illustrative purposes.

We set out to investigate the molecular mechanisms of nanobody-induced complement inhibition in greater detail. We first assessed whether the nanobodies could prevent the formation of C4b2. To this end, C4b and C2 were incubated in the presence of nanobodies and analyzed by SEC to evaluate the impact of nanobodies on C4b2 formation (Fig. 2). NbB5, NbE11, and NbH9 prevented the formation of the proconvertase, because we observed only C2 and C4b nanobody peaks (Fig. 2A–C). In contrast, in the presence of NbG3, the elution profile resembled that of C4b2 (Fig. 2D), indicating that NbG3 did not prevent proconvertase formation, even though it is an inhibitor in the lysis assay. Similarly, NbB12 did not affect the formation of C4b2 (Fig. 2E), which agrees with the lack of inhibition of this nanobody. Interestingly, the elution profiles in the presence of NbE3 showed only partial proconvertase assembly, as indicated by a broadened peak between C4b2 and C4b and half depletion of the C2 peak (Fig. 2F). Similarly, the presence of NbH11 yielded a reduced level of proconvertase (Fig. 2G). Thus, these results showed that NbG3 and NbB12 have no effect on C4b2 formation and that NbB5, NbE11, NbH9, and to a lesser extent NbH11 and NbE3 inhibit proconvertase formation.

### C4b inactivation by C4BP and FI in the presence of nanobodies

We next tested the ability of C4BP and FI to generate C4c and C4d (inactive C4b fragments) by cleaving the C4b α′-chain in the presence of nanobodies. NbB5, NbH11, and NbE3 prevented C4b cleavage, as shown by the presence of intact C4b α′-chain (87 kDa) (Fig. 2H). Nanobodies NbB12, NbE11, NbG3, and NbH9 did not block C4c and C4d generation, as indicated by bands observed at 75, 25, and 17 kDa of the β, α3, and α4 chains, respectively, of C4c and at 47 kDa of C4d. However, when the cofactor concentration was increased five times, only NbB5 effectively prevented C4b cleavage (Supplemental Fig. 2H). These data suggest that NbE3 and NbH11 hinder, whereas NbB5 blocks, C4BP or FI binding to C4b, and NbB12, NbE11, NbG3, and NbH9 do not interfere with cofactor activity.

### Cryo-EM structure determination of C4b–nanobody complexes

**FIGURE 3.**
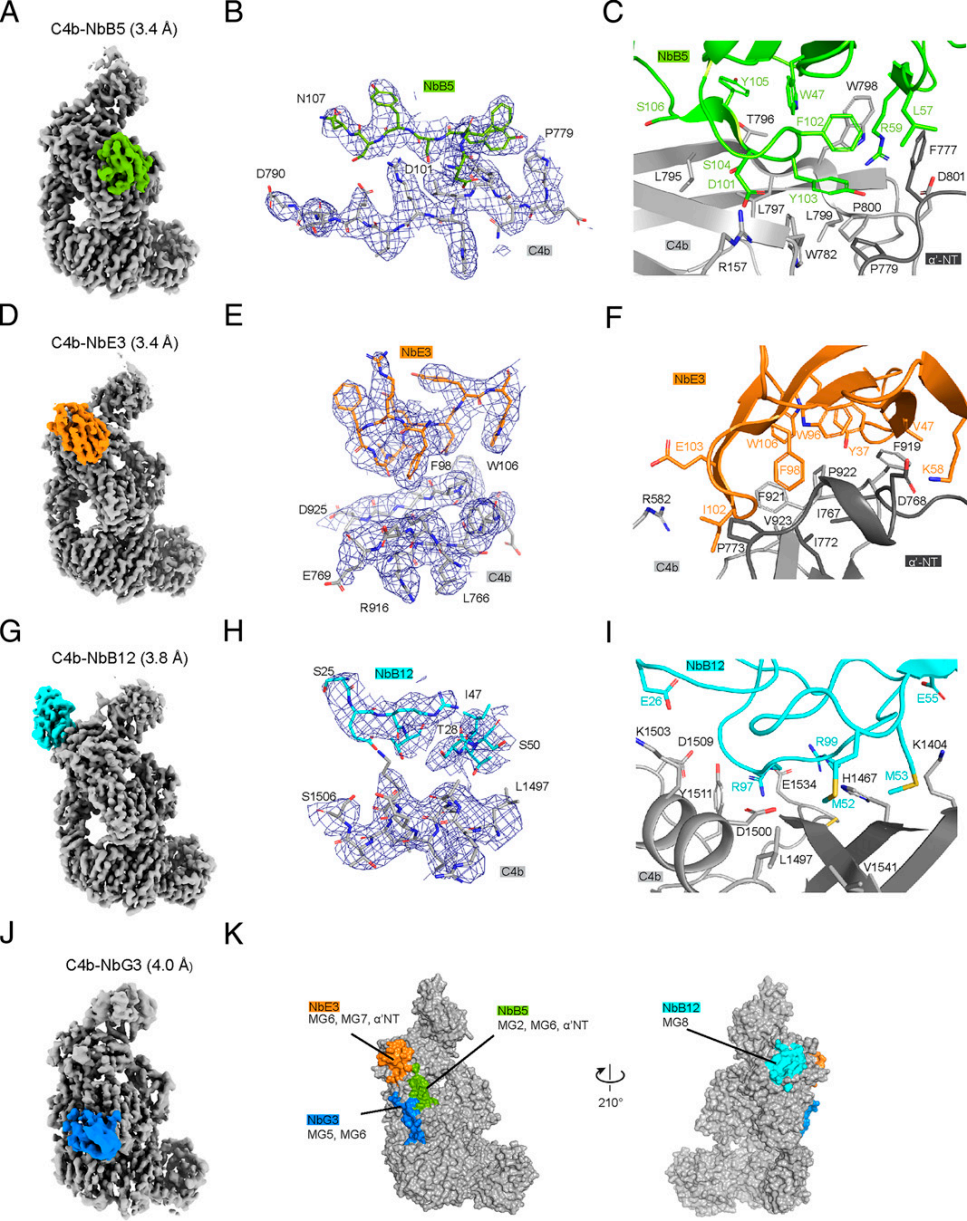
Cryo-EM structures of C4b in complex with nanobodies. (**A**–**J**) Cryo-EM density maps post-processed using DeepEMhancer (61) and structural details of C4b (gray) in complex with NbB5 (green, A–C), NbB12 (cyan, D–F), Nb-E3 (orange, G–I), and NbG3 (blue, J). Modeled amino acids of selected regions of the structures of C4b in complex with (B) NbB5, (E) NbE3, and (H) NbB12 shows a part of the C4b–Nb interface. C4b–nanobody structures shown as zoom of the binding interface of C4b with (C) NbB5, (F) NbE3, and (I) NbB12 in the same colors introduced in (A), (D), and (G). Amino acid residues that likely contribute to the interaction are annotated and shown as sticks. (**K**) Surface representation of C4b (PDB accession no. 5JTW) in two orientations of C4b represented as surface with nanobody footprints. The C4b surface that is within 4 Å of nanobodies is colored according to the Nb colors previously assigned.

To rationalize the diverse functional effects observed with four of our C4b-binding nanobodies, we determined structures of C4b–nanobody complexes using single-particle cryo-EM of four distinct nanobodies: NbB5, NbB12, NbE3, and NbG3. Micrographs showed well-distributed protein particles of homogeneous size in vitreous ice for C4b-NbB5, C4b-NbE3, and C4b-NbB12, whereas severe particle clustering was observed for C4b-NbG3. Image processing in RELION yielded 2D class averages, which displayed clear secondary structure elements and showed features of nanobody-bound C4b (Supplemental Fig. 3A). Detailed processing resulted in 3D cryo-EM density maps at global resolutions of 3.4 Å for C4b-NbB5 and C4b-NbE3, 3.8 Å for C4b-NbB12, and 4.0 Å for C4b-NbG3 (Fig. 3A–J, Table II, Supplemental Fig. 3). In the case of C4b-NbG3, the map was highly distorted and anisotropic, which is likely caused by strong preferred orientation and clustering of the C4b-NbG3 particles in the vitreous ice layer. Although the quality of this map did not allow sidechain modeling, density for NbG3 and consequently its binding location on C4b could be clearly distinguished (Fig. 3J, K). The C4b-NbB5, C4b-NbB12, and C4b-NbE3 maps showed sufficient details for modeling of sidechains (Fig. 3B, E, H), and the structures were built using a previously reported structure of C4b (PDB accession no. 5JTW) and SWISS-MODEL generated nanobody structures as starting models. The refined structures of the three C4b–nanobody complexes exhibit acceptable model statistics and stereochemistry (Table II).

**Table II.** Cryo-EM data collection, refinement, and validation statistics

	C4b-NbB5 PDB 7B2P, EMDB-11989	C4b-NbE3 PDB 7B2M, EMDB-11988	C4b-NbB12 PDB 7B2Q, EMDB-11990	C4b-NbG3 EMDB-11991
Data collection and processing				
Microscope	Talos Arctica	Talos Arctica	Talos Arctica	Talos Arctica
Camera	Gatan K2 Summit + GIF	Gatan K2 Summit + GIF	Gatan K2 Summit + GIF	Gatan K2 Summit + GIF
Magnification	130,000	130,000	130,000	130,000
Voltage (kV)	200	200	200	200
Exposure time frame/total (s)	0.2/7.2	0.2/6.4	0.2/6.4	0.2/6.4
Number of frames	36	32	32	32
Electron exposure (e^-^/ Å^2^)	51.7	51.7	53.9	50.5
Defocus range (µm)	−0.8 to −3.0	−0.8 to −3.0	−0.8 to −3.0	−0.8 to −3.0
Pixel size (Å)	1.029	1.029	1.029	1.029
Micrographs (no.)	1,032	1,396	1,453	1,658
Initial particle images (no.)	522,079	617,600	602,481	845,087
Final particles images (no.)	274,925	176,318	128,161	207,769
Map resolution (Å) 0.143 FSC threshold	3.43	3.39	3.76	3.96
Map resolution range (Å)	3.3–5.7	3.3–5.1	3.6–5.7	3.8–6.3
Refinement				
Model resolution (Å) 0.5 FSC threshold	3.50	3.43	3.86	
Map sharpening B factor (Å^2^)	−125	−72	−100	
Model composition				
Nonhydrogen atoms	11,690	11,664	11,612	
Protein residues	1,509	1,506	1,495	
Ligands	NAG: 5	NAG: 4	NAG: 5	
B factors (Å^2^)				
Protein	71.1	69.1	75.5	
Ligands	87.9	81.9	81.4	
r.m.s.d.				
Bond lengths (Å)	0.003	0.003	0.003	
Bond angles (°)	0.520	0.489	0.541	
Validation				
MolProbity score	1.78	1.59	1.85	
Clashscore	8.12	6.41	9.07	
Rotamer outliers (%)	0.0	0.0	0.0	
Ramachandran plot				
Favored (%)	95.12	96.44	94.70	
Allowed (%) Outliers (%)	4.88 0.00	3.56 0.00	5.23 0.07	

r.m.s.d., root mean square deviation.

The observed architecture of C4b in the nanobody-bound cryo-EM structures is similar to previously published crystal structures of C4b (19, 40), indicating that crystal packing did not greatly impact the conformation of the protein. A notable difference with the crystal structures is the poor cryo-EM density for the CTC domain (Fig. 3A, D, G, J), which did not allow detailed modeling. This suggests that the CTC orientation is variable when it is not stabilized by crystal packing or additional protein interactions, which is in agreement with previous small-angle x-ray scattering studies (19) and cryo-EM data acquisition on C4 homolog C5, where the CTC domain also resulted in a 5–6 Å local map resolution (66).

### Binding interfaces

Overall, nanobodies NbB5, NbE3, and NbB12 displayed similar C4b-binding paratope architectures with a central hydrophobic pocket stabilized by surrounding electrostatic interactions (Fig. 3C, F, I). The binding of NbB5 and NbE3 involved residues belonging to the nanobody framework region and CDR2 and CDR3. Both nanobodies interact with adjacent regions on the C4b α′-NT and neighboring domains (Fig. 3C, F), whereas for NbB12, CDR1, 2, and 3 are responsible for its interaction with C4b (Fig. 3I).

The observed interface between C4b and NbB5 buries a total surface area of 1692 Å^2^. NbB5 binds in a pocket between two β-sheet regions of the MG6 domain of C4b (Fig. 3C). NbB5-CDR3 residues Y103, S104, Y105, and S106 adopt a β-strand conformation and form a short, antiparallel β-sheet with C4b-MG6 residues L795, T796, L797, and W798. The sidechains of F102, Y103, and Y105 of CDR3 and W782, W798, L799, and P800 of the MG6 domain form a hydrophobic core of the C4b–NbB5 interface, which is further composed of W48 and L57 of CDR2 and F777 and P779 of the α′-NT. The periphery of the interface is stabilized by several polar interactions, including salt bridges between D101 in CDR3 and R157 from MG2 and R59 from the nanobody framework region 3 and MG7 residue D801. Besides interacting in a salt bridge, NbB5 residue R59 is sandwiched between C4b residues F777 and W798, thereby forming potential cation-π stacking interactions.

The C4b–NbE3 interface observed in the structure buries 1689 Å^2^ surface area. Similar to the interface with NbB5, the C4b–NbE3 interface contains a hydrophobic pocket, formed by NbE3-Y37, V47, W96, F98, I102, and W106 from framework region 2, CDR2, and CDR3 (Fig. 3F) and with C4b-I767, I772, and P773 from the α′NT and F919, F921, P922, and V923 of MG7. The polar interactions that surround the hydrophobic pocket compose a salt bridge between K58 from nanobody framework region 3 and D768 in the α′NT and E103 in CDR3 with R582 in MG5, as well as additional hydrogen bonding interactions.

Even though the C4b-NbB12 map (at 3.8 Å resolution) allowed modeling, the nanobody region was of lower quality when compared with the C4b-NbB5 and C4b-NbE3 maps. The lower quality is likely caused both by the lower resolution of the C4b-NbB12 map and a stronger preferred orientation of the C4b-NbB12 particles (Supplemental Fig. 3C). We therefore interpret the interface between C4b and NbB12 with caution. The interface buried a surface area of 1460 Å^2^. The C4b-binding epitope of NbB12 comprises three β-strands and a short α-helix of the MG8 domain (Fig. 3I). The interface is formed by all three CDRs of NbB12, and each CDR of NbB12 is involved in electrostatic interactions with C4b. These electrostatic interactions include salt bridges between C4b-K1503 and CDR1-E26, K1404 and CDR2-E55, and E1534 and CDR3-R97 and R99. NbB12 forms hydrophobic contacts with the backbone of C4b-R1465–H1467 through CDR2-M53, whereas the neighboring CDR2-M52 interacts with C4b-L1497 and V1541. Other C4b residues that contribute to the interactions are D1500, D1509, Y1511, C1535, and G1537.

Finally, the anisotropic density map of C4b-NbG3 did not allow characterization of the binding interface at a sidechain level. However, the binding location of NbG3 was clearly defined by rigid body fitting of C4b and NbG3 models into the map, revealing that NbG3 binds C4b in its β-ring area through interactions with the MG5-MG6 linker and the MG6 domain. Thus, the structural data allowed structural determination of the binding site of all four nanobodies at a domain level and for NbB5, NbB12, and NbE3 at a residue level.

### NbB12 functionalized for labeling in in vitro studies

**FIGURE 4.**
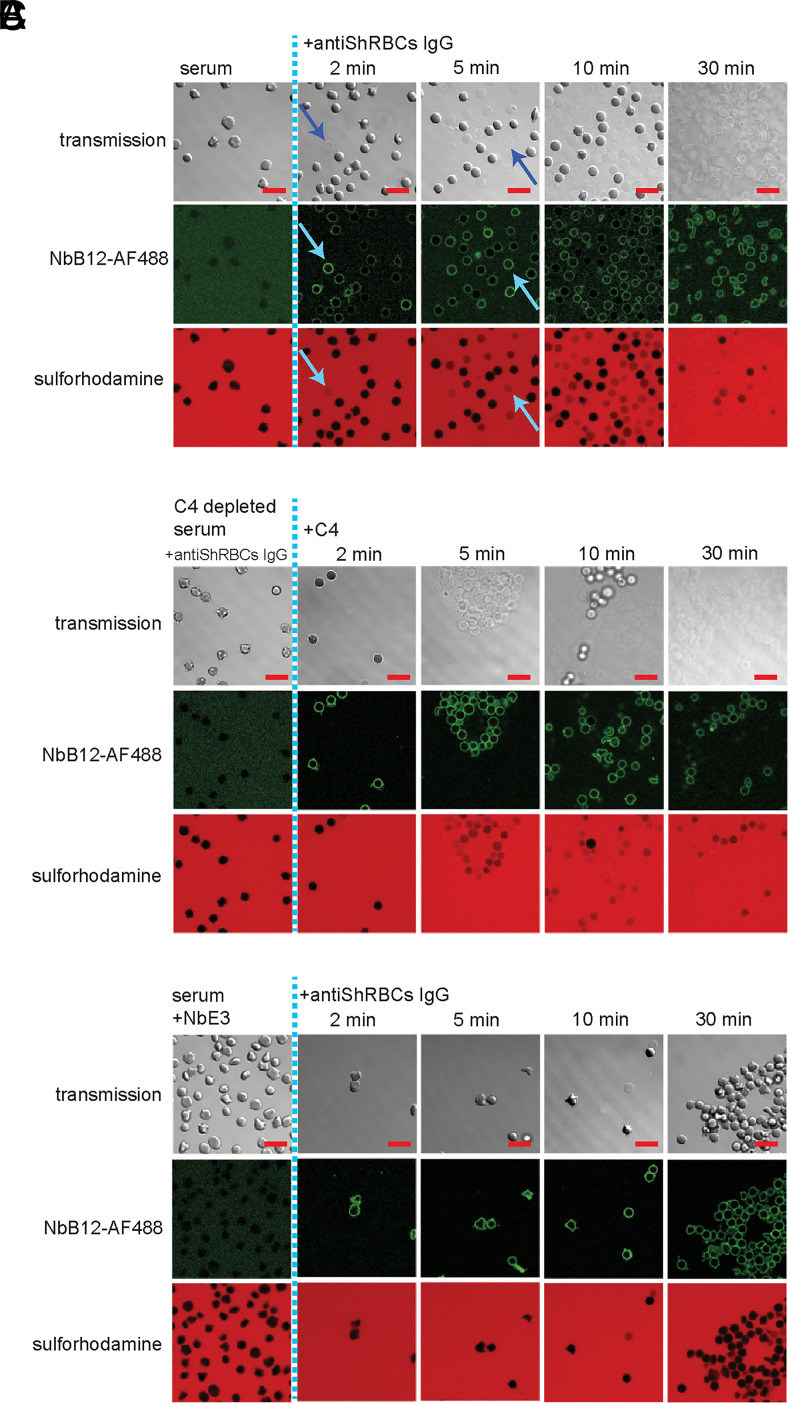
Functionalization of NbB12 as an in vitro fluorescent probe (NbB12–Alexa Fluor 488) to follow complement activation on SRBCs by confocal microscopy. Deposition and lysis of erythrocytes was followed in the transmission channel, in green fluorescence (to monitor C4b deposition, denoted by binding of NbB12–Alexa Fluor 488), and in red fluorescence to monitor influx of sulforhodamine B into the cell upon complement lysis activated by anti-SRBCs IgG in (**A**) normal human serum. Blue arrows indicate the correlation between channels in cell permeabilization by loss of contrast in the transmission channel, abundant C4b deposition on the NbB12–Alexa Fluor 488 channel, and influx of sulforhodamine B in the red channel. (**B**) C4-depleted serum, reactivated by addition of purified C4 and (**C**) normal human serum in presence of strong inhibitor NbE3.

**FIGURE 5.**
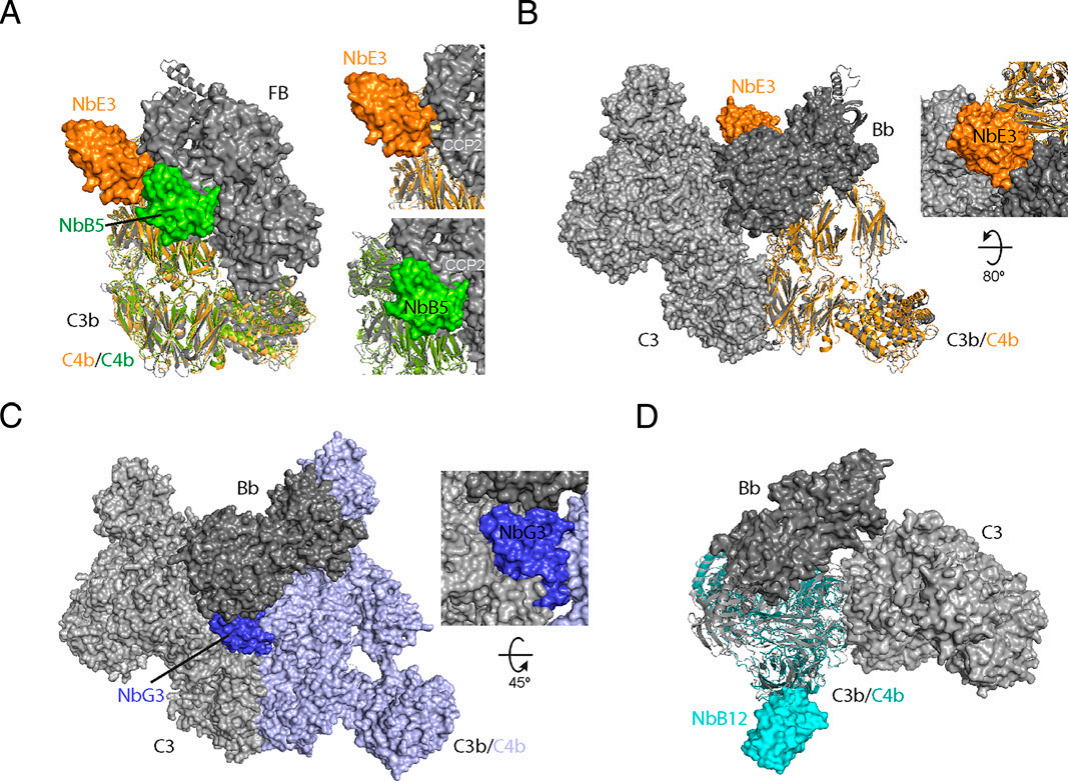
Superpositions of C4b–nanobody structures onto C3b proconvertase and convertase structures. (**A**) Superposition of the structures of C4b-NbB5 (green) and C4b-NbE3 (orange) onto C3b-B (PDB accession no. 2XWJ, gray), with C3b and C4b shown in cartoon drawing and NbB5, NbE3, and factor B in surface representation; insets highlight contacting domains. (**B**–**D**) C4b–nanobody structures superposed onto a structural model of C3b-Bb in complex with C3 that is based on dimers of C3bBb inhibited by SCIN (PDB accession no. 2WIN), with C3b in gray cartoon representation, Bb in dark gray, and C3 in gray surface representation. C4b-NbE3 is shown in orange, with C4b in ribbon and NbE3 in surface representation (B). For C4b-NbG3, C4b is shown in surface representation (light blue), and NbG3 is indicated in dark blue (C). C4b-NbB12 is shown in cyan, with C4b in cartoon and NbB12 in surface representation; (D) is in top view relative to (B) and (C).

NbB12 did not affect complement activation or regulation in our assays (Figs. 1B–E, 2E, and 2H). Therefore, we termed it as a “silent” nanobody. We next investigated whether fluorescently labeled NbB12 could be used as a tool for monitoring membrane deposition of C4b during CP initiation. To test this, we activated CP on SRBC membranes through human serum and anti-SRBC IgG in the presence of fluorescently labeled NbB12 (NbB12–Alexa Fluor 488). Upon complement activation, membranes displayed green fluorescence, indicating binding of NbB12–Alexa Fluor 488 to membrane-deposited C4b (Fig. 4A–C). High NbB12–Alexa Fluor 488 binding signal at initial stages (2–5 min) of complement activation correlated with influx of sulforhodamine B into SRBCs and a loss of contrast in the transmission channel (indicated by blue arrows in (Fig. 4A), likely due to the release of cytoplasmic material to the surroundings. After 30 min of incubation, nearly all cells were lysed (Fig. 4A), in agreement with results from the hemolysis assay (Fig. 1B, D). Replacing normal human serum by C4-depleted serum prevented permeabilization, and no NbB12–Alexa Fluor 488 binding to the cell membranes was observed (Fig. 4B). However, supplementing the reaction mixture after 15 min with purified C4 at equivalent serum concentration restored both processes to levels observed with normal human serum (Fig. 4B), pointing out the critical role for C4 in these processes. We further tested the efficacy of NbE3 as a CP inhibitor. In the presence of NbE3, human serum and anti-SRBC IgG, immediate binding of NbB12–Alexa Fluor 488 to the erythrocytes was observed. Nevertheless, complement lysis was minimal after 30 min of incubation (Fig. 4C), whereas in the absence of NbE3, extensive permeabilization of cells was observed after a few minutes of incubation (Fig. 4A). Consistent with our previous observations, NbE3 acts as a strong inhibitor. Although NbE3 binding allows C4 activation and C4b deposition, it interferes with the complement cascade toward pore formation (Fig. 5).

## Discussion

We presented seven nanobodies against human C4b that showed either no (NbB12), moderate (NbG3 and NbH9), or strong (NbB5, NbE3, and NbH11) CP inhibition. The remaining nanobody (NbE11) indicated weak inhibition (i.e., observed only at the lower two serum concentrations used), but for this case, the data were statistically not conclusive. Five of seven nanobodies (NbB5, NbB12, NbBE3, NbH9, and NbH11) bind C4b with higher affinity than C4, whereas two (NbE11 and NbG3) bind both C4 and C4b.

Four nanobodies in complex with C4b were selected for cryo-EM structure determination: three inhibitors with distinct activities (NbB5, NbE3, and NbG3) and one silent binder (NbB12). Cryo-EM density maps were reconstructed at resolutions of 3.4–4.0 Å. The reconstructed cryo-EM maps of C4b-NbB5, C4b-NbB12, and C4b-NbE3 allowed model building. The density map of the inhibitor C4b-NbG3 was too anisotropic for an accurate model building; however, the nanobody density clearly identified the binding site on C4b. The epitopes for the nanobodies are formed by residues of domains MG2, MG6, and α′NT region for NbB5; MG8 for NbB12; MG6, MG7, and α′NT for NbE3; and the MG5-MG6 linker region and MG6 for NbG3 (Fig. 3K). Upon nanobody binding, no large conformational changes in C4b were observed in any of the four structures when compared with C4b crystal structures (19, 40, 57). Consistent with preferred binding to C4b and not C4 (Supplemental Fig. 2C, F), NbB5 and NbE3 interact with the C4b α′NT region (Fig. 3C, F) that relocates and becomes exposed upon cleavage of C4 into C4b. The only other reported C4b nanobody, hC4Nb8, binds C4b at a position similar to NbB5, interacting with MG6 and α′NT, and exhibits reduced affinity toward C4 (40). Furthermore, the binding site of NbB12, formed by residues in MG8, is hidden by the C4a domain in C4 and hence explains its C4b binding preference (Supplemental Fig. 2E). Last, NbG3 binds domain MG6 and its connection to MG5 (Fig. 3K). This region is structurally equivalent in C4 and C4b, which explains why NbG3 binds both C4 and C4b (Supplemental Fig. 2A). Thus, the structural data rationalize the observed C4-C4b binding preferences of the four nanobodies.

To understand the modes of action of the anti-C4b nanobodies NbB5, NbB12, NbE3, and NbG3, we compared the C4b nanobody structures with structures of the AP proconvertase and convertase formed by C3b and factor B (in the absence of relevant complexes of C4b and C2; (Fig. 5). Superposing C4b-NbB5 onto C3bB, PDB accession no. 2XWB, shows that the potent inhibitor NbB5 coincides with domain CCP2 of factor B (Fig. 5A), suggesting that NbB5 most likely blocks C2 binding to C4b. This is consistent with our observation that NbB5 prevents the formation of the CP proconvertase (Fig. 2A). Similarly, the reported hC4Nb8 (40) exhibits an overlapping epitope with NbB5 and blocks proconvertase formation (Supplemental Fig. 4A). Superposing C4b-NbE3 onto C3bB shows that binding of NbE3 is adjacent to but does not overlap with factor B binding (Fig. 5A). The ^768^DEDD cluster of C4b forms part of the NbE3 epitope (Fig. 3F), which is reported to form part of the C2 binding site (23). Our data indicate that NbE3 partially blocks proconvertase formation (Fig. 2F); nevertheless, NbE3 strongly inhibits CP-mediated lysis (Figs. 1B–E, 5C). Comparison of the C4b–nanobody complexes with an AP convertase substrate model (i.e., C3bBb-C3, based on structural dimers of C3bBb-SCIN; PDB accession no. 2WIN [26]) suggests that NbE3 and NbG3 most likely block C3 substrate binding (Fig. 5B, C). This notion is further supported by comparable C3b–inhibitor complexes. NbE3 binds C4b (at domain MG7 and α′NT) similar to how Ab S77 and nanobody hC3NB1 bind to C3b (Supplemental Fig. 4B) (38, 67), whereas NbG3 binding to C4b (at domain MG6 and its linker to MG5) can be compared with CRIg binding to C3 molecules (Supplemental Fig. 4C) (39, 68). These AP inhibitors prevent proconvertase formation and block substrate binding to the convertase (38, 67, 68). Finally, the binding site of the silent nanobody, NbB12, is located at the MG8 domain that is directly involved in neither (pro)convertase formation nor convertase activity (Fig. 5D), hence explaining its noninhibitory activity. In summary, nanobody binding to the observed sites on C4b explains the inhibitory activity of NbB5, NbE3, and NbG3 and the “silent” binding of NbB12.

The observed lack of, or reduced, formation of C4c and C4d fragments by cofactor C4BP and FI due to inhibitors NbB5 and NbE3 (Fig. 2H), respectively, can be understood by comparison of C4b to structural homolog C3b, in complex with FH and FI (PDB accession no. 2WII [35] and PDB accession no. 5O32 [37]). All complement cofactor activity and decay acceleration regulators bind C3b on a transverse platform along the α′NT, MG7, MG6, MG2, MG1, CUB, and TED (36), which is likely homologous to C4b regulator binding (69–72). Superposing C3b-FH domain 1-4 (35) onto the C4b-NbB5 structure indicates that binding of NbB5 would clash with CCP2 domain binding of C4BP (equivalent to CCP2 of FH binding to C3b; Supplemental Fig. 4D), thereby blocking cofactor interaction. NbE3 binds C4b at MG7 and α′NT in a position that blocks FH-CCP1 binding to C3b (Supplemental Fig. 4D). Blocking C4BP at this position hinders C4b degradation, but it does not fully abolish it (Supplemental Fig. 2H); this is consistent with the minor contribution of CCP1 to the C3b-FH domain 1-4 interface. In a similar way, these nanobodies would hinder binding of C4BP, and possibly CR1 and DAF, to C4b to exert decay acceleration activity on the CP convertase. In contrast to NbB5 and NbE3, inhibitor NbG3 did not affect C4c and C4d formation at the tested concentrations (Fig. 2H, Supplemental Fig. 2H), which can be explained by distinct binding sites for NbG3 and cofactors. Thus, although NbB5, NbE3, and the reported hC4Nb8 (40) block C4b degradation, NbG3 permits C4c and C4d formation. Therefore, potential therapeutic application of these inhibiting nanobodies likely differs.

For three nanobodies, NbE11, NbH9, and NbH11, no structural data are available, and their modes of action cannot be fully determined. NbE11 clearly prevented proconvertase formation using purified proteins (Fig. 2B). However, its inhibition of complement activation in the lysis was minimal (Fig. 1B, C). Although NbE11 and NbH9 have partially overlapping C4b binding sites (Supplemental Fig. 1Q), NbH9 prevented proconvertase formation and inhibited complement activation in the lysis assay strongly. The only other difference observed was that NbE11 binds more readily to C4 than NbH9 does, whereas both bind C4b equally well. Because C4 undergoes large conformational rearrangements upon activation into C4b, there must be a distinct difference in their precise epitopes, even though the two epitopes overlap in part. How the apparent binding to C4 of NbE11 is related to its very weak inhibition is unclear. The C4b binding site of NbH11 overlaps with that of NbB5 (Supplemental Fig. 1I), for which the structure was determined in complex with C4b. Both nanobodies exhibit almost identical C4b affinities (Table I), reduce proconvertase formation, and inhibit complement activation in the lysis assay (Fig 1B). At high cofactor concentration, NbB5 still prevents C4b degradation, whereas NbH11 does not block C4c and C4d formation. Possibly, NbH11 overlaps less with the cofactor binding site than NbB5, which would explain its similar inhibition of proconvertase formation and complement activation and relatively reduced inhibition of C4c and C4d formation.

In conclusion, we presented seven antihuman C4b nanobodies suitable for research and in vitro assays to study complement activation of the CP and putatively of the LP. A noninhibiting “silent-binder” nanobody has potential for imaging of complement activation. Finally, the other six nanobodies offer several possibilities for potential diagnostic and therapeutic applications to study and modulate the activation of the CP of complement, which has been linked to immunological and neuronal pathologies.

## Supplementary Material

Data Supplement
