# Supplementary material for: Multifaceted Activities of Seven Nanobodies against Complement C4b
Source: J Immunol. 2022 May 1;208(9):2207–19. doi: 10.4049/jimmunol.2100647 (PMC9047069; doi:10.4049/jimmunol.2100647)
Supplement: Data Supplement [file JI_2100647.zip › JI_2100647_Supplemental_1.pdf]

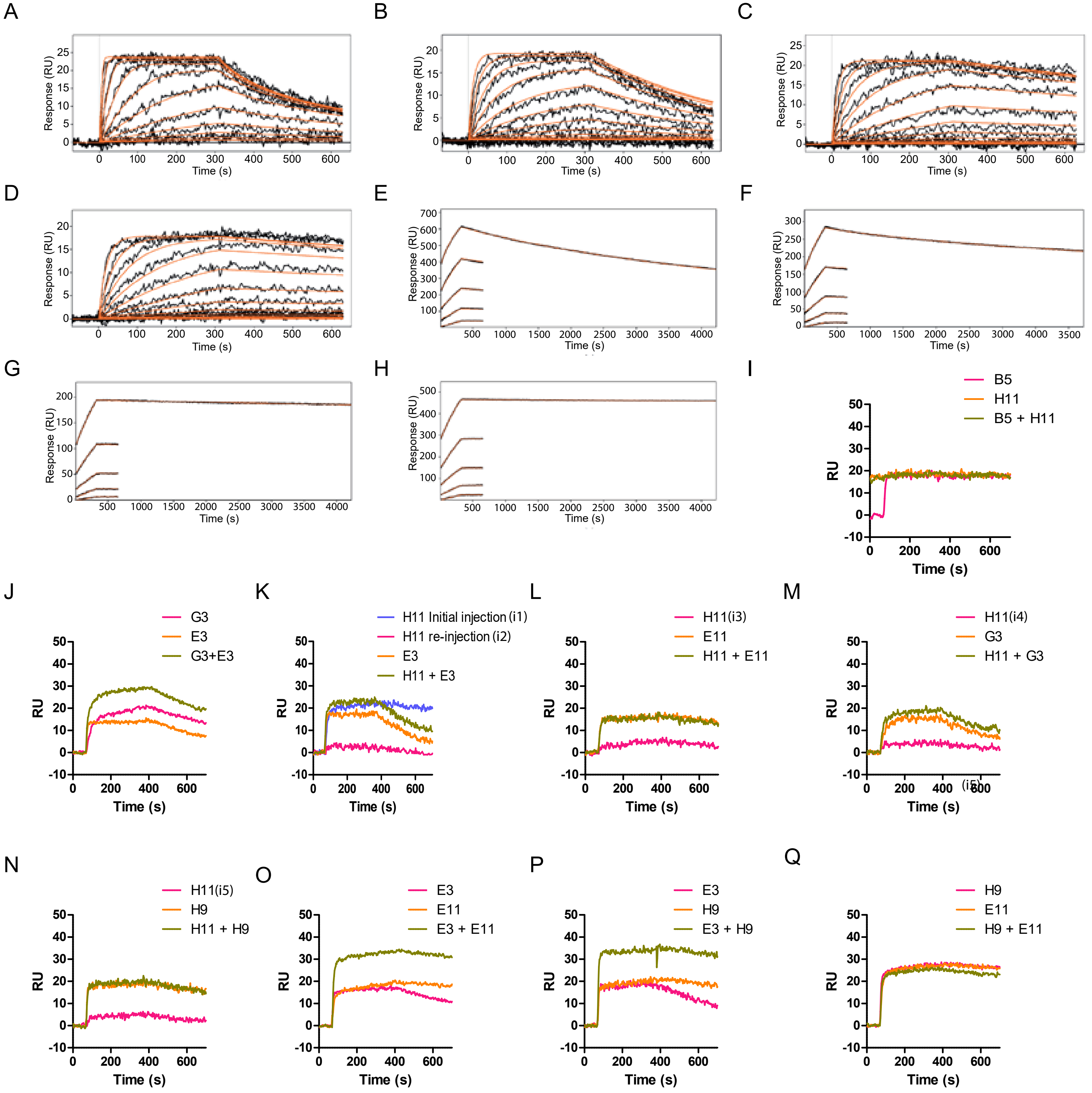

Fig. S1: SPR binding curves for affinity determination and binding competition assays between nanobodies to determine overlapping binding sites on C4b.

Panels A- D show SPR curves for nanobodies A) NbG3, B) NbE3, C) NbH9 and D) NbE11 with C4b as a ligand and the nanobody as the analyte evaluated during 600 seconds with analyte concentrations of 0.78, 1.56, 3.13, 6.25, 12.5 and 25 nM. SPR binding curves E-H, show nanobodies E) NbE11, F) NbB5, G) NbH11 and H) NbB12 as a ligand and C4b as the analyte evaluated over 4000 seconds with analyte concentrations of 0.20, 0.39, 0.78, 1.56, 3.13 and 6.25 nM. Panels I- Q) show one-to-one competition binding curves between nanobodies. C4b was used as a ligand and the nanobodies as analytes (100 nM). Each curve shows two independent injections of a single nanobody (first injection in pink and second injection in orange) followed by a single injection with both nanobodies (green). For panel K) the chip was saturated with an initial injection of NbH11 marked as injection 1 (i1), shown in blue. Due to little NbH11 dissociation the following injections of NbH11 were labelled consecutively, as the chip was still saturated with NbH11. Injections of NbH11 are labelled i2, i3, i4 and i5 shown in pink for panels K, L, M and N, respectively. Increase of relative units (RU) for NbE3, NbE11, NbG3 and NbH9 single injections (orange) or in combination with NbH11 denote mutual binding with NbH11. For panels I, J, O, P and Q, increase in RU on the combined injections curve (green) compared to single nanobody injections denote mutual binding. However, equal units to single nanobody injections indicate epitope competition

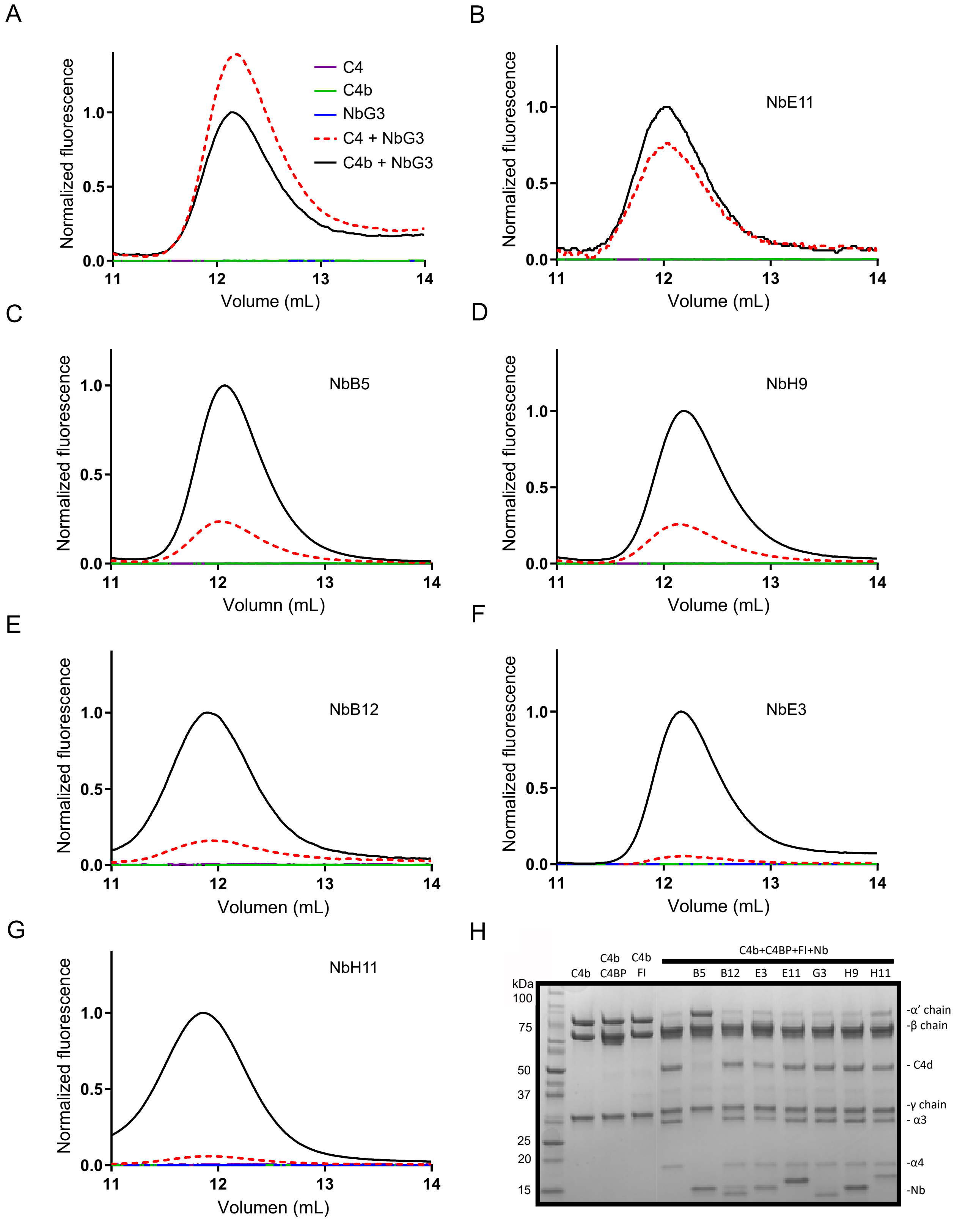

Fig S2. Size-exclusion chromatography elution profiles of C4 and C4b in complex with fluorescent-tagged nanobodies and C4b inactivation cleavage evaluated by SDS-PAGE.
Fluorescent signal at C4 or C4b elution volumes (around 12 mL) denoted formation of C4 and C4b-nanobody complexes for A) NbG3, B) NbE11, C) NbB5, D) NbH9, E) NbB12 F) NbE3 and G) NbH11. The fluorescence of the complexes was normalized to the C4b-nanobody signal (black) with C4-nanobody complexes shown in red (dotted line). In addition, C4b (purple), C4 (green) and the nanobody (blue) profiles are shown as controls. Panel H) shows C4BP and FI-mediated cleavage of C4b in presence or absence of nanobodies. SDS PAGE shows the disappearance of the C4b α'chain (87 kDa) and the formation of products C4d (47 kDa), α3 (25 kDa) and α4 (17 kDa) after incubation of 1 h at 37 °C in molar ratios 1:0.5:0.02:2 of C4b:C4BP:FI:nanobody, respectively. C4BP concentration was increased five-fold compared to assay shown in Fig. 2H.

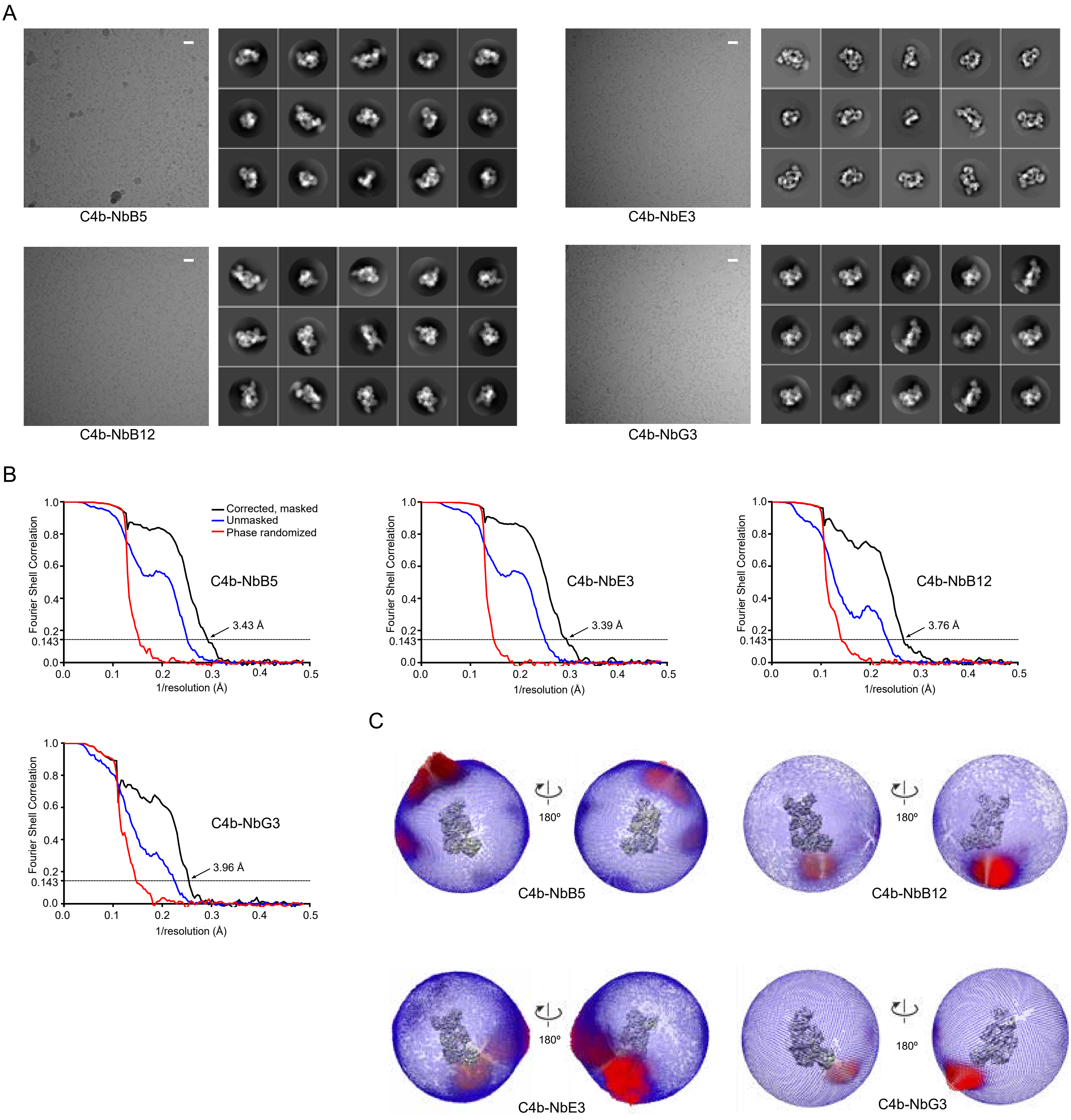

Fig S3: Cryo-EM image processing of the C4b-nanobody datasets.
A) Micrograph in vitreous ice of C4b-nanobody particles and selected 2D-class averages for NbB5, NbE3, NbB12 and NbG3, respectively. The scale bar length is 200 Å. B) Fourier-shell correlation plots for the gold-standard refined C4b-nanobody reconstructions, computed from masked (black), unmasked (blue) and high-resolution phase-randomized (red) half maps. C) Angular distribution of the C4b-nanobody particles that yielded the final 3D reconstruction, shown in two different orientations.

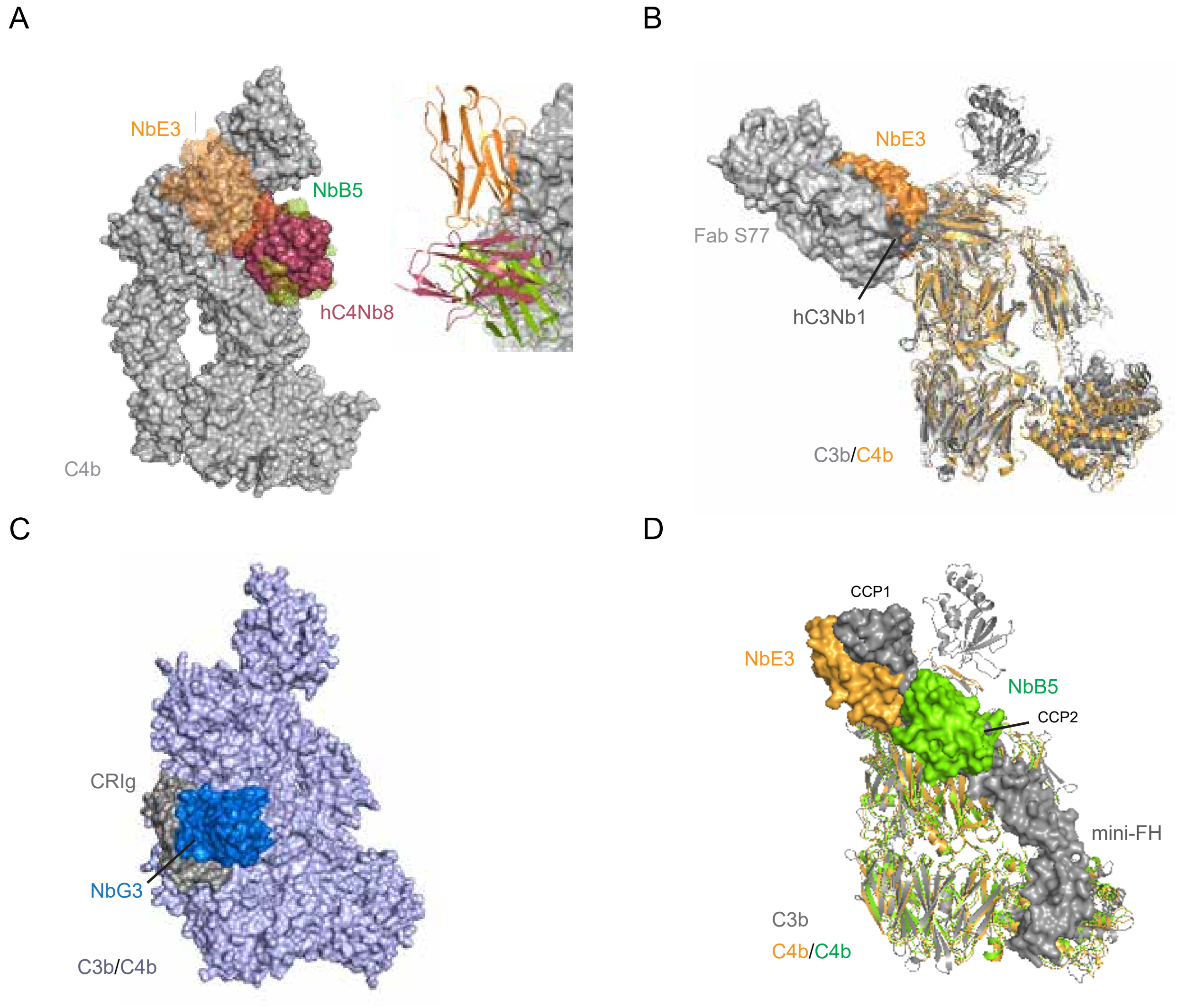

Fig S4: Superposition of C4b-nanobody structures and AP C3 convertase inhibitors and regulator factor H domain 1-4.
A) Superposition of the structures of C4b-NbB5 (green), C4b-NbE3 (orange) and C4b-hC4Nb8 (pdb 6YSQ, dark red). NbE3 and NbB5 are shown in cartoon as well as semitransparent surface and hC4Nb8 and C4b (grey) are shown in surface representation. Zoom-in panel shows all nanobodies in cartoon representation. B) C4b-NbE3 structure (orange) superposed to C3b structures with inhibitors of the AP, C3b-hC3Nb1(pdb 6EHG, dark grey) and C3b-S77 Fab (pdb 3G6J, light grey), where C4b and C3b structures are shown in cartoon and NbE3, hC3Nb1 and S77 Fab are shown in surface. (C) Structure of C4b (pdb 5JTW) fitted into C4b-NbG3 density map was superposed with C3b-CRIg structure (pdb 2ICF), where C4b and C3b are shown in light blue, NbG3 in blue and CRIg in dark grey, all shown as surface representation. D) Superposition of C4b-NbB5 (green) and C4b-NbE3 (orange) onto the structure of C3b bound to factor H domain 1-4 (pdb 2WII, grey). All C3b and C4b structures are shown in cartoon and NbE3, NbB5 and mini factor H are shown as a surface representation.
